# Microstructural Optimization and Erosion–Corrosion Resistance of Cu-10Ni-3Al-1.8Fe-0.8Mn Alloy via Tailored Heat Treatment

**DOI:** 10.3390/ma18071511

**Published:** 2025-03-27

**Authors:** Yi Yuan, Yizhi Zhao, Yicheng Cao, Lue Huang, Hao Chu, Hongqian Wang, Dongyan Yue, Wenjing Zhang

**Affiliations:** 1 State Key Laboratory of Nonferrous Structural Materials, China GRINM Group Co., Ltd., Beijing 100088, China; 18975151855@163.com (Y.Y.); caoyicheng1995@163.com (Y.C.); mse.frozen@gmail.com (H.C.); hxixi719@126.com (H.W.); willimyue@foxmail.com (D.Y.); 2 GRIMAT Engineering Institute Co., Ltd., Beijing 101407, China; 3 General Research Institute for Nonferrous Metals, Beijing 100088, China; 4 School of Materials Science and Engineering, South China University of Technology, Guangzhou 510640, China; yizhizhao1994@scut.edu.cn (Y.Z.); huangluehl@163.com (L.H.)

**Keywords:** copper–nickel alloy, heat treatment, erosion–corrosion, Ni_3_Al

## Abstract

This study systematically investigated the effects of tailored heat treatments on the microstructural evolution, mechanical properties, and erosion–corrosion resistance of Cu-10Ni-3Al-1.8Fe-0.8Mn alloy. Four heat treatment conditions—as-cast (AC-1); homogenized (H-2); and deformation–aged at 500 °C (D-3) and 750 °C (D-4)—were applied to elucidate the interplay between microstructure and performance. The D-3 specimen, subjected to deformation followed by aging at 500 °C for 0.5 h, demonstrated superior properties: a Vickers hardness of 118 HV5 (83.3% higher than H-2) and an erosion–corrosion rate of 0.0075 mm/a (84.1% reduction compared to H-2). These enhancements were attributed to the uniform dispersion of nanoscale Ni_3_Al precipitates within the matrix, which optimized precipitation strengthening and reduced micro-galvanic corrosion. The D-3 specimen also formed a dense, crack-free Cu_2_O corrosion product film with a flat matrix interface, confirmed by SEM cross-sectional analysis and electrochemical impedance spectroscopy (EIS), exhibiting the highest charge transfer resistance and film impedance.

## 1. Introduction

Copper–nickel alloys exhibit outstanding corrosion-resistance properties, particularly in marine applications, rendering them critical components in key industrial applications, including shipbuilding, offshore structural engineering, and seawater desalination systems [1,2,3]. However, the harsh conditions of seawater, such as high salinity, dissolved oxygen, and mechanical erosion, present substantial durability challenges to these alloy systems [4,5]. To enhance the performance of copper alloys in marine environments, researchers have optimized their chemical compositions and microstructures through various heat treatments and deformation processes [6,7]. Ma et al. [8] demonstrated that grain boundary engineering can significantly improve the erosion–corrosion performance of 90Cu10Ni tubes. Alloying elements can improve the performance of copper–nickel alloys in erosive marine applications. Ni enhances the corrosion resistance and oxidation resistance of the alloy [9,10], while Al helps form a protective oxide layer [11,12,13]. Fe improves the mechanical properties of the alloy through both solid solution strengthening and the formation of intermetallic phases [14,15], while Mn acts synergistically with Fe [16]. It was found that copper–nickel alloys with an added Al content of 3 wt% had the lowest corrosion rate after 10 days of immersion in artificial seawater [17]. However, the corrosion behavior of this alloy under the synergistic conditions of mechanical impact and electrochemical corrosion, such as erosion–corrosion phenomena, remains insufficiently investigated [18]. Therefore, on the basis of the above studies, we designed a Cu-10Ni-3Al-1.8Fe-0.8Mn alloy and optimized its microstructure, mechanical properties, and erosion–corrosion performance by different heat treatments.

This study systematically investigated the effects of various heat treatment processes on the Cu-10Ni-3Al-1.8Fe-0.8Mn alloy, focusing on its microstructure evolution, mechanical properties, and erosion–corrosion behavior. Four distinct heat treatment conditions were employed: as-cast (AC-1), homogenized (H-2), deformed and aged at 500 °C (D-3), and deformed and aged at 750 °C (D-4). These treatments were designed to explore the interplay between microstructure and performance, particularly the role of precipitation hardening and the formation of protective oxide layers. This study aims to provide a comprehensive understanding of how heat treatment processes can be optimized to enhance the alloy’s performance in demanding marine environments by examining these aspects. The findings from this research are expected to contribute to the development of advanced materials with improved mechanical and corrosion-resistant properties, suitable for high-demand applications in harsh conditions.

## 2. Experimental

### 2.1. Materials

Alloy with the composition of Cu-10Ni-3Al-1.8Fe-0.8Mn was prepared by melting pure copper, nickel, aluminum, Cu-20Fe master alloy, and manganese in a vacuum induction furnace. The resulting ingot had a diameter of Φ63 mm. The chemical composition of the prepared alloy is shown in Table 1. The alloy ingot was homogenized at 950 °C for 4 h and then hot-extruded to a diameter of Φ24 mm, followed by cold drawing with a deformation of 15%. Finally, the alloy was quenched after continuous aging for 30 min at 500 °C and 750 °C, respectively. The alloy specimens treated under four different heat treatments were designated as AC-1, H-2, D-3, and D-4, as shown in Table 2.

### 2.2. Methods

#### 2.2.1. Mechanical Properties Testing

The hardness of the specimens was measured at room temperature using a Vickers hardness tester (WILSON VH1150, Chicago, IL, USA). Before measurement, the surface of the specimens was polished with sandpaper up to 2000# to remove surface oxides and other impurities that might affect the hardness measurement, and to facilitate accurate readings. A load of 5 kg was applied to the specimens for a dwell time of 15 s. After unloading, a square indentation was left on the specimen, and the length of the diagonal of the indentation was measured to calculate the corresponding Vickers hardness value. Each specimen was measured at least five times, and the average value was taken as the result.

#### 2.2.2. Erosion–Corrosion Testing

**Figure 1:**
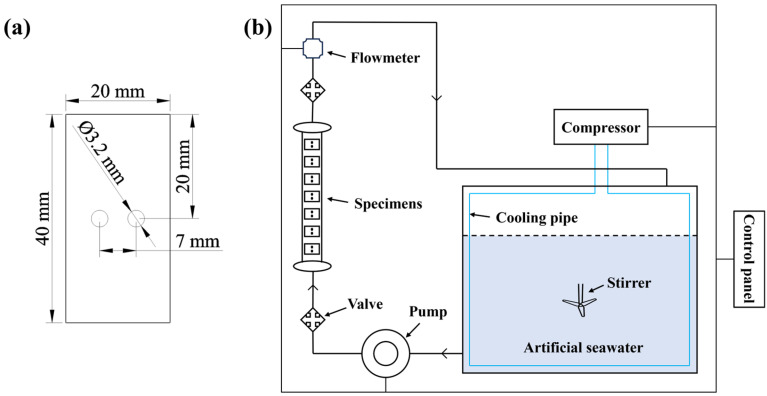
(**a**) Dimensions of the erosion–corrosion specimens. (**b**) Schematic diagram of the self-made erosion–corrosion testing pipeline apparatus.

To simulate real marine corrosion environments, erosion–corrosion tests were conducted on four specimens. For each one, four parallel samples with dimensions of 20 mm × 40 mm × 2 mm were prepared, as shown in Figure 1a. After polishing, cleaning, and drying, the samples were weighed and placed into a self-made erosion–corrosion apparatus (Figure 1b). The apparatus consists of a water tank, a water pump, and a control unit, with the flow velocity regulated by adjusting the frequency of the water pump. The composition of the artificial seawater used in the test was as follows: NaCl (24.530 g/L); MgCl_2_ (5.200 g/L); Na_2_SO_4_ (4.090 g/L); CaCl_2_ (1.160 g/L); KCl (0.695 g/L); NaHCO_3_ (0.201 g/L); KBr (0.101 g/L); H_3_BO_3_ (0.027 g/L); SrCl_2_ (0.025 g/L); and NaF (0.003 g/L).

During the erosion–corrosion test, the temperature was controlled at 30 ± 2 °C, the flow velocity of the artificial seawater was maintained at 2 m/s, the pH value was around 7.8, and the dissolved oxygen content was approximately 7 mg/L. The test duration was 7 days. After the test, the samples were cleaned in hydrochloric acid solution for 3 min, then removed, cleaned with alcohol, and dried. They were weighed again using an analytical balance with a precision of 0.0001 g to measure the mass loss of each sample. The erosion–corrosion rate was calculated using the following formula [19]:

(1) $$R=\frac{8.76\times 10^{7}\times (M-M_{1}-M_{k})}{\mathrm{STD}}$$

In the formula, R is the erosion–corrosion rate (mm/a); M is the mass of the specimen before the test (g); M_1_ is the mass of the specimen after the test (g); M_k_ is the average mass loss of the blank specimens (g); S is the total surface area of the specimen (cm^2^); T is the test duration (h); and D is the density of the specimen (kg/m^3^). The results of the erosion–corrosion test reflect the performance of the alloy in a dynamic corrosive environment, highlighting the combined effects of mechanical impingement and chemical attack on the material [18].

#### 2.2.3. Microstructural Characterization

Microstructural characterization was conducted using a field-emission electron probe microanalyzer (EPMA, JXA-iHP200F, JEOL, Tokyo, Japan) to observe the microstructure and elemental distribution in the matrix of AC-1 and H-2 specimens. The samples were prepared by cutting, grinding, and polishing, and they were then used for EPMA testing, with SEM images and elemental analysis results collected.

A field-emission transmission electron microscope (TEM, FEI Titan Cube 80-300, Madison, WI, USA) was employed to characterize the microstructure of the matrix in D-3 and D-4 specimens. The samples were thinned to a thickness of 50–60 µm, punched into circular discs with a diameter of 3 mm, and subsequently subjected to electrochemical thinning. The grain structure, grain boundaries, and precipitates were observed using TEM, and diffraction and EDS analyses were performed.

A scanning electron microscope (SEM, JEOL, JSM, 7200F, Tokyo, Japan) was used to examine the surface and cross-sectional morphology of the corrosion product films formed on the four specimens after 7 days of erosion–corrosion testing in artificial seawater. The morphology and structure of the corrosion product films were compared and analyzed. The distribution of elements in the corrosion product films was determined using EDS analysis.

#### 2.2.4. EIS Testing

Electrochemical impedance spectroscopy (EIS, VersaSTAT 4, AMETEK, Berwyn, PA, USA) tests were conducted on the four specimens after 7 days of erosion–corrosion in artificial seawater to compare and analyze the impedance of the corrosion product films formed on each specimen, thereby evaluating the protective nature of these films against the underlying alloy matrix. The tests were performed using a three-electrode system with a frequency range from 100 kHz to 0.01 Hz and an AC excitation signal amplitude of 5 mV.

## 3. Results and Discussion

### 3.1. Microstructure

**Figure 2:**
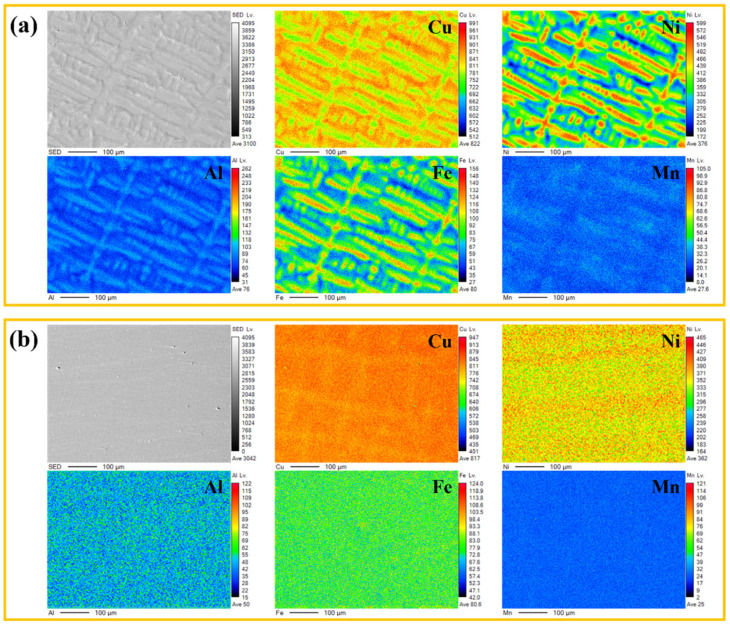
EPMA elemental maps of the microstructures of the specimens: (**a**) AC-1 and (**b**) H-2.

Figure 2 shows the EPMA elemental maps of the matrix microstructures of the AC-1 and H-2 specimens. As shown in Figure 2a, the matrix of the AC-1 specimen contained many dendritic structures, with significant inhomogeneity in the distribution of Cu, Ni, and Fe elements. This indicated severe elemental segregation in the as-cast alloy. Such segregation commonly occurs during casting processes, primarily due to significant differences in elemental diffusion rates during solidification, leading to non-uniform distribution of elements between dendrites and dendrite trunks. Particularly, Fe and Mn elements, with their low diffusion rates, tended to accumulate in the interdendritic regions, forming localized segregation zones [20,21]. In contrast, the H-2 specimen, which had undergone homogenization heat treatment at 950 °C for 4 h (Figure 2b), exhibited significantly improved elemental distribution uniformity. The homogenization treatment, conducted through prolonged isothermal holding at elevated temperatures, facilitated elemental diffusion and effectively mitigated segregation in the as-cast structure. EPMA mapping revealed improved uniformity in Cu, Ni, and Fe distributions, demonstrating the treatment’s efficacy in enhancing microstructural homogeneity. Additionally, homogenization treatment helped eliminate residual stresses in the as-cast structure, providing a favorable microstructural basis for subsequent deformation and aging processes. Notably, no obvious precipitates were observed in the matrix of either specimen. It was suggested that in the as-cast and homogenized states, second-phase particles had not yet significantly precipitated in the alloy.

**Figure 3:**
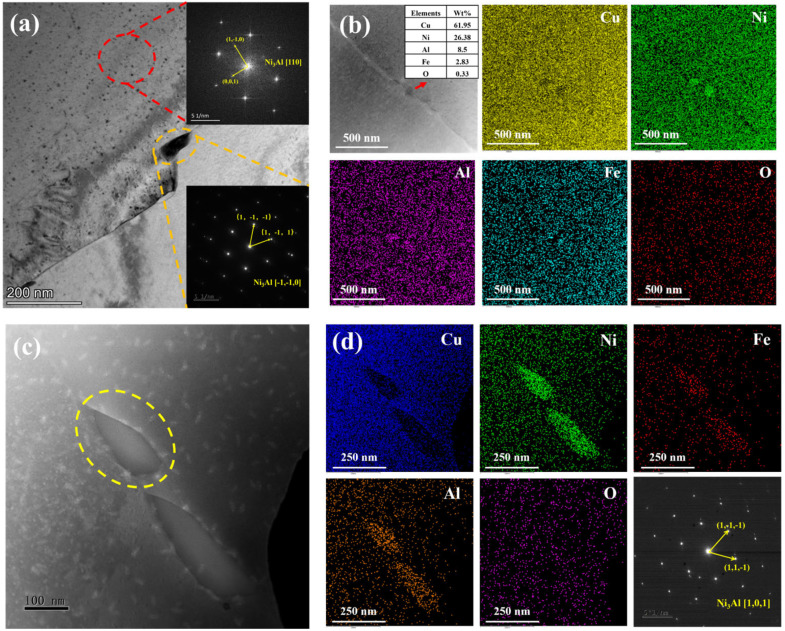
TEM images of the microstructures of the specimens: (**a**,**b**) D-3 and (**c**,**d**) D-4.

Figure 3 shows the TEM images of the D-3 and D-4 specimens, corresponding to the microstructures after different aging treatments based on the homogenized state (D-3: 500 °C/0.5 h; D-4: 750 °C/0.5 h). Figure 3a displays the microstructure of the D-3 specimen and the corresponding diffraction spots. Fine precipitates with a size of approximately 100 nm were observed along the grain boundaries, while numerous dispersed phases existed within the grains. Through diffraction spot analysis, it was determined that the fine dispersed phases within the grains and the precipitates on the grain boundaries were both Ni_3_Al, corresponding to the [110] and [$\bar{1}\bar{1}0$] zone axes, respectively. Further EDS analysis (Figure 3b) revealed that the precipitates on the grain boundaries were rich in Ni, Fe, and Al. This indicated that under the aging treatment at 500 °C, Ni_3_Al preferentially nucleated and grew at the grain boundaries. Meanwhile, Fe segregated at the grain boundaries, partially substituting Ni atoms in Ni_3_Al to form (Ni, Fe)_3_Al.

Figure 3c displayed the microstructure of the D-4 specimen, with discontinuously distributed precipitates of approximately 200 nm in size along the grain boundaries. To further analyze the composition and structure of these precipitates, EDS mapping of the region in Figure 3c and diffraction analysis of the circled precipitates were performed (Figure 3d). The results indicated that the precipitates in the D-4 specimen were also of the Ni_3_Al structure but contained higher Fe content, corresponding to the [101] zone axis. When compared to the D-3 specimen, the D-4 specimen exhibited coarsened precipitates with complete absence of intragranular dispersed phases. This was because at the higher aging temperature of 750 °C, the atomic diffusion rate increased significantly, causing the precipitates to grow rapidly and form coarse grain boundary precipitates. Meanwhile, the nucleation and growth of precipitates within the grains were suppressed.

The differences in precipitates between the D-3 and D-4 specimens were mainly attributed to the different aging temperatures. At the lower aging temperature of 500 °C, although the driving force for precipitation was large, the atomic diffusion rate was relatively slow. The precipitates tended to nucleate at the grain boundaries and grow slowly, forming fine dispersed phases. In contrast, at the higher aging temperature of 750 °C, the atomic diffusion rate increased significantly, allowing the precipitates to grow rapidly and form coarse grain boundary precipitates within a short time. Additionally, the segregation behavior of Fe elements at the grain boundaries was also affected by the aging temperature. At higher temperatures, Fe was more likely to enrich at the grain boundaries and form (Ni, Fe)_3_Al with the Ni_3_Al.

### 3.2. Mechanical Properties

**Figure 4:**
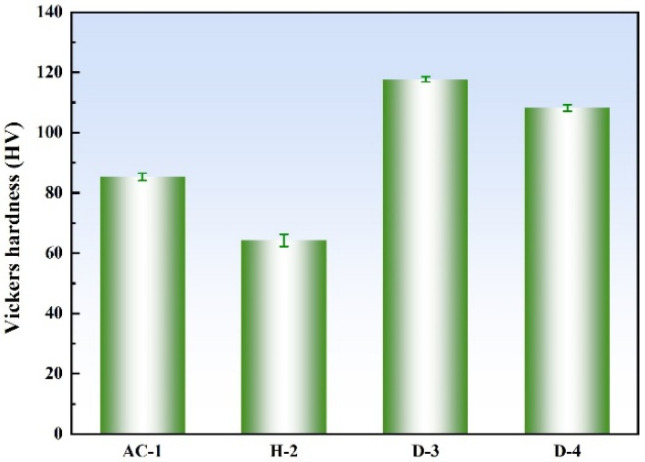
Vickers hardness of the four specimens under different heat treatments.

Figure 4 shows the Vickers hardness test results of the four specimens under different heat treatments. As can be seen from the figure, the D-3 specimen exhibited the highest hardness, reaching 118 HV5, while the H-2 specimen had the lowest hardness, at only 64 HV5. The hardness of the D-3 and D-4 specimens was higher than that of the AC-1 specimen. This was attributed to the homogenization treatment and deformation processing they underwent, as well as the precipitation of secondary phases in the matrix after aging treatment. These processes led to solid solution strengthening, work hardening, and precipitation strengthening, significantly enhancing their mechanical properties.

The highest hardness of the D-3 specimen was primarily due to the dispersed Ni_3_Al in its microstructure. According to the Orowan mechanism, dispersed precipitates can effectively hinder dislocation movement, thereby significantly increasing the material’s hardness [22]. In the D-3 specimen, Ni_3_Al within the grains was small in size, and (Ni, Fe)_3_Al at the grain boundaries was approximately 100 nm in size. Although grain boundary precipitates can impede grain boundary sliding and dislocation movement, their strengthening effect is generally weaker than that of fine and dispersed precipitates within the grains. Therefore, the high hardness of the D-3 specimen was mainly attributed to the significant precipitation strengthening provided by dispersed Ni_3_Al within the grains, while the small size of the grain boundary precipitates further contributed to the hardness.

The D-4 specimen had a lower hardness than the D-3 specimen but was still higher than that of the AC-1 specimen. This was because, at the higher aging temperature of 750 °C, the precipitates tended to grow rapidly, forming larger grain boundary precipitates (approximately 200 nm), while inhibiting the nucleation and growth of fine and dispersed precipitates within the grains. Although grain boundary precipitates can provide some strengthening effect, their larger size makes them less effective in hindering dislocation movement, resulting in the lower hardness of the D-4 specimen compared to the D-3 specimen.

The lowest hardness of the H-2 specimen was mainly due to the high-temperature homogenization treatment at 950 °C. This process increased the grain size and released residual stresses in the as-cast structure, leading to a more uniform distribution of alloying elements and eliminating the local high-hardness regions caused by dendritic segregation in the as-cast structure. Additionally, no precipitation strengthening occurred during the homogenization treatment, resulting in significantly lower hardness for the H-2 specimen compared to the D-3 and D-4 specimens that underwent aging treatment.

### 3.3. Corrosion Resistance

**Figure 5:**
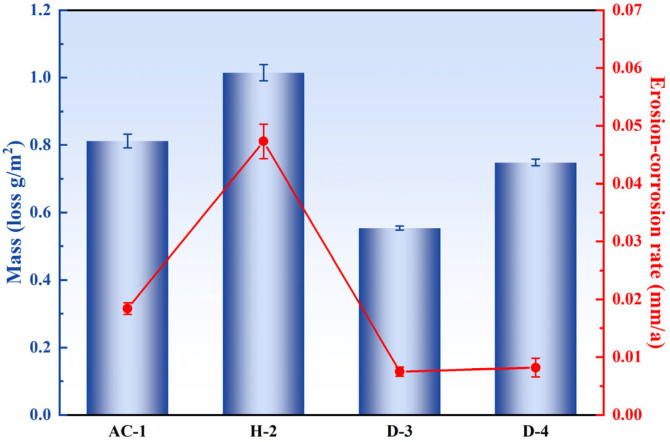
Mass loss and erosion–corrosion rate of the four specimens after 7 days of erosion–corrosion in artificial seawater under different heat treatments.

Figure 5 shows the mass loss and erosion–corrosion rates of the four specimens subjected to different heat treatments after 7 days of erosion–corrosion in artificial seawater. The D-3 specimen exhibited the lowest erosion–corrosion rate of 0.0075 mm/a, demonstrating excellent corrosion resistance. In contrast, the AC-1 and H-2 specimens had higher erosion–corrosion rates and poorer corrosion resistance.

The enhanced corrosion resistance of the D-3 specimen principally originated from the uniform dispersion of Ni_3_Al particles within its microstructure. This phase reduced the potential difference between anodic and cathodic regions, thereby diminishing the driving force for localized electrochemical corrosion. The Ni_3_Al phase, acting as a stable cathodic phase, attracted electrons from the corrosive medium, reducing the anodic dissolution rate of the matrix material and significantly lowering the corrosion rate [23,24]. Additionally, the presence of the dispersed phase hindered the diffusion paths of the corrosive medium through grain boundaries and phase boundaries, slowing down the diffusion rate of corrosive species. The dispersed phase also reduced local stress concentrations in the material, lowering the risk of stress corrosion cracking (SCC). The excellent mechanical properties of the D-3 specimen enabled it to better withstand the impingement of artificial seawater, reducing mechanical damage to the material surface and further enhancing its corrosion resistance.

The corrosion resistance of the D-4 specimen was slightly inferior to that of the D-3 specimen, mainly due to the larger size of precipitates at the grain boundaries, which facilitated the formation of micro-galvanic cells with the matrix, leading to selective corrosion [25,26]. Although the D-4 specimen had better mechanical properties to resist impingement, its corrosion rate remained higher than that of the D-3 specimen. The poorer corrosion resistance of the AC-1 and H-2 specimens was mainly due to severe elemental segregation and compositional inhomogeneity in the as-cast structure, which exacerbated localized electrochemical corrosion. Moreover, the H-2 specimen experienced grain coarsening during homogenization treatment, further reducing its corrosion resistance.

### 3.4. Corrosion Product Film

**Figure 6:**
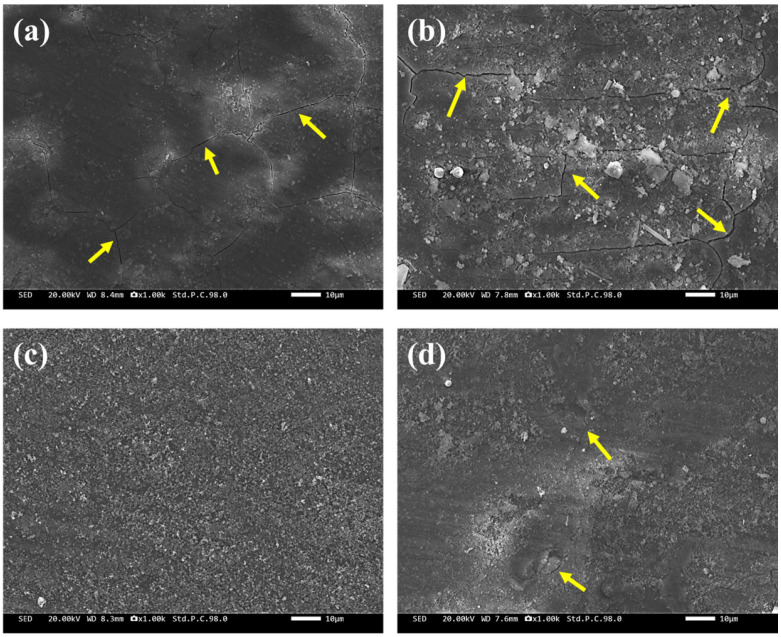
Surface morphology of the corrosion product films on the four specimens after 7 days of erosion–corrosion in artificial seawater under different heat treatments: (**a**) AC-1, (**b**) H-2, (**c**) D-3, and (**d**) D-4.

Figure 6 shows the surface morphology of the corrosion product films formed during 7-day erosion–corrosion testing in artificial seawater, with arrows indicating the locations of film cracking. Observations using SEM revealed significant differences in the surface structures of the corrosion product films among the different specimens. For the H-2 specimen, the surface of the corrosion product film exhibited numerous cracks, which were caused by shear stresses during the erosion process or by the corrosive attack of the medium. These cracks allowed the corrosive medium to penetrate more easily into the matrix, thereby accelerating the corrosion process and reducing the protective nature of the film. In stark contrast, the corrosion product film on the D-3 specimen showed almost no cracks and exhibited a much more intact and dense structure. This indicated that the passivation film on the D-3 specimen effectively resisted the impingement of artificial seawater and the penetration of corrosive species, providing better protection for the underlying alloy matrix. The corrosion product films on the AC-1 and D-4 specimens also contained some cracks, but their quantity and size were smaller than those on the H-2 specimen.

**Figure 7:**
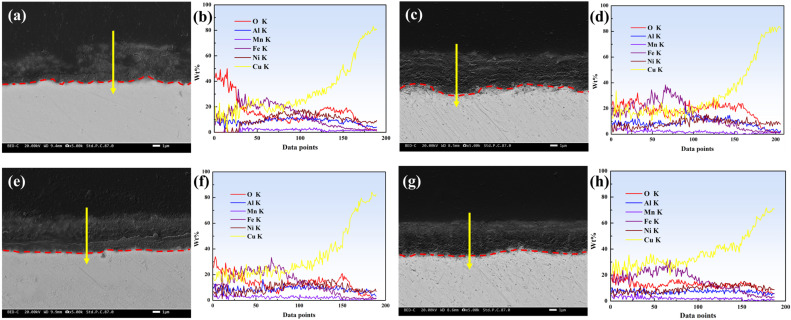
Cross-sectional morphology of the corrosion product films and EDS line-scan results of the films after 7 days of erosion–corrosion in artificial seawater under different heat treatments: (**a**,**b**) AC-1, (**c**,**d**) H-2, (**e**,**f**) D-3, and (**g**,**h**) D-4.

Figure 7 shows the cross-sectional morphology of the corrosion product films and the EDS line-scan results for the specimens. The cross-sectional observations further elucidate the interfacial structure between the corrosion product film and the matrix, as well as the elemental distribution within the film. Structurally, the interface between the corrosion product film and the matrix in the D-3 specimen was very flat, indicating that the matrix of the D-3 specimen exhibited good resistance to impact during the erosion–corrosion process in artificial seawater, maintaining a relatively smooth surface. This flat interface structure helps reduce stress concentration, thereby enhancing the adhesion and protective nature of the film. Moreover, the EDS line-scan results revealed that the main constituents of the corrosion product films in all four specimens were oxides, with O and Cu elements distributed relatively uniformly within the films. The O-to-Cu ratio was close to 1:2, indicating that the primary component of the films was Cu_2_O. Cu_2_O is a common protective oxide whose dense structure effectively blocks the penetration of corrosive species. However, the protective performance of the corrosion product film is not solely determined by its chemical composition; it is also closely related to its structural integrity, thickness, and adhesion to the matrix.

Overall, the corrosion product film of the D-3 specimen exhibited the densest structure and the flattest interface, demonstrating the best protective performance. This was attributed to its superior mechanical properties, as well as the dispersed Ni_3_Al within the grains, acting as a cathodic phase. Ni_3_Al formed micro-galvanic couples with the matrix, promoting passivation of the matrix. The high chemical stability of Ni_3_Al meant that it did not readily dissolve during corrosion, providing a stable base for the formation of the corrosion product film and facilitating the uniform growth and densification of Cu_2_O. This further enhanced the corrosion resistance of the alloy.

### 3.5. EIS Analysis

**Figure 8:**
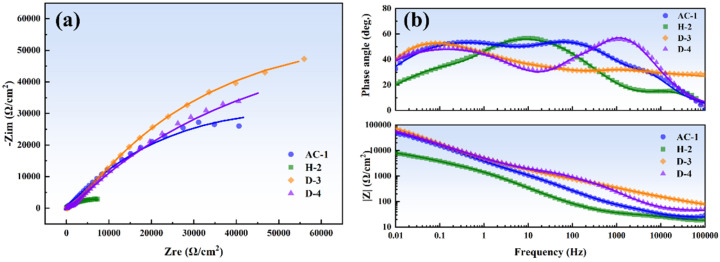
EIS test results of the four specimens after 7 days of erosion–corrosion in artificial seawater under different heat treatments: (**a**) Nyquist plot and (**b**) Bode plot.

Figure 8 shows the EIS results of the four specimens after 7 days of erosion–corrosion in artificial seawater under different heat treatments. The Nyquist plot indicates that all specimens exhibited a distinct semicircle in the high-frequency region, which is associated with the charge transfer process at the electrode/electrolyte interface. The diameter of the semicircle reflects the magnitude of the charge transfer resistance (R_ct_). The D-3 specimen had the largest semicircle diameter, indicating the highest R_ct_, likely due to its dense corrosion product film that slowed down the interfacial reaction kinetics. In contrast, the H-2 specimen had the smallest semicircle diameter, indicating the lowest R_ct_ and the fastest interfacial reaction kinetics. None of the specimens showed a clear Warburg impedance characteristic in the low-frequency region, suggesting that their electrochemical processes were not diffusion-controlled. The impedance in the low-frequency region was primarily dominated by the corrosion product film. Consistently, the D-3 specimen exhibited the highest impedance in the low-frequency region, indicating the highest resistance of its corrosion product film and better protective performance.

The phase angle curve in Figure 8b shows that the D-3 specimen had a broader phase angle peak, indicating a larger interfacial capacitance. The impedance modulus (|Z|) curve revealed that all specimens had a relatively low impedance modulus in the high-frequency region, dominated by the electrolyte resistance (R_s_). The D-3 specimen had the highest impedance modulus in the low-frequency region, significantly larger than that of the H-2 specimen. The Bode plot results further supported the analysis from the Nyquist plot, confirming that the D-3 specimen had the highest impedance of the corrosion product film and the best protective properties.

**Figure 9:**
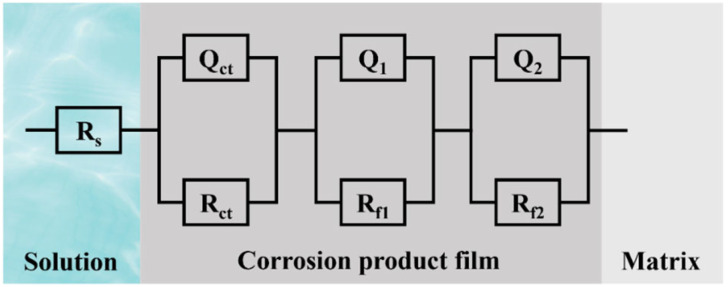
Equivalent circuit for EIS analysis.

Figure 9 illustrates the equivalent circuit model for EIS analysis: R(QR)(QR)(QR). Here, R_s_ represents the resistance of the electrolyte, i.e., the resistance of artificial seawater, which appears as the impedance value in the high-frequency region (usually on the far-left side of the Nyquist plot). Q_ct_ represents the double-layer capacitance at the electrode/electrolyte interface. Since real interfaces are not ideal capacitors and exhibit non-ideal behaviors (such as surface roughness, inhomogeneity, and porosity), using a constant phase element (CPE) to describe this non-ideal capacitive behavior can improve the accuracy of the equivalent circuit fitting, especially when the corrosion product film has a multi-layer structure or complex characteristics. The mathematical expression for CPE is as follows [27]:

(2) $$Z_{CPE}=\frac{1}{Y_{0}{\left(j\omega \right)}^{n}}$$

where $Y_{0}$ is the conductance parameter of the CPE, and n is the exponent of the CPE (0 ≤ n ≤ 1). When n = 1, the CPE behaves as an ideal capacitor; when n = 0, the CPE behaves as an ideal resistor. A value of n close to 1 indicates that the interface or film is close to an ideal capacitor, while a smaller value of n indicates higher non-uniformity of the interface or film. R_ct_ is the charge transfer resistance, representing the resistance to charge transfer at the electrode/electrolyte interface and reflecting the kinetic barrier of the interfacial reaction. The larger the R_ct_, the slower the charge transfer process. The (Q_ct_R_ct_) combination describes the charge transfer process at the electrode/electrolyte interface, typically corresponding to the high-frequency semicircle in the Nyquist plot. Q_1_ and Q_2_ represent the capacitive behavior of different layers or different characteristics of the corrosion product film. *R_f_*_1_ and *R_f_*_2_ represent the resistances of different layers or different characteristics of the corrosion product film, reflecting the impedance of the corrosion product film to ion migration. The (Q_1_R_f1_) and (Q_2_R_f2_) combinations correspond to the mid-low frequency region in the Nyquist plot.

Table 3 lists the fitting parameters of the EIS equivalent circuit. *R_t_* is the total resistance, calculated by the following formula [28]:

(3) $$R_{t}=R_{ct}+R_{f1}+R_{f2}$$

By comparing the values, it was found that the D-3 specimen had the highest *R_ct_*, *R_f_*_1_, and *R_f_*_2_, indicating that its corrosion product film was the densest and provided the best protection for the underlying matrix. Additionally, its Q_ct_ and Q_2_ were relatively large, and the values of n_1_, n_2_, and n_3_ were close to 1, suggesting that its interface and corrosion product film were close to ideal capacitors and had a more compact structure. In contrast, the corrosion product film of the H-2 specimen had a more porous and loose structure, resulting in the poorest protective performance.

### 3.6. Mechanism of Corrosion Product Film Formation

**Figure 10:**
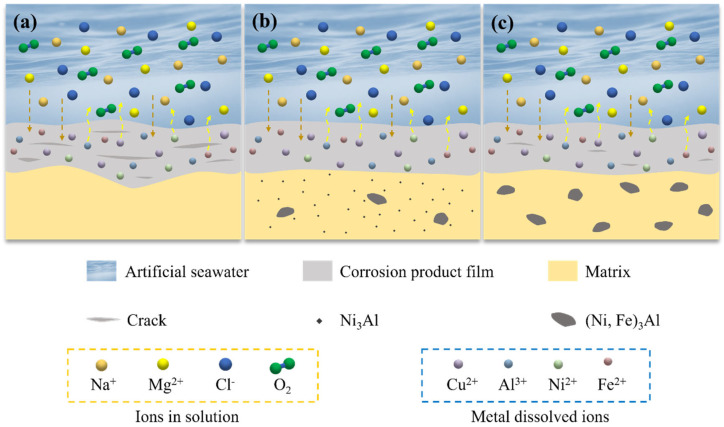
Schematic illustration of the mechanism of corrosion product film formation: (**a**) AC-1 and H-2; (**b**) D-3; and (**c**) D-4.

The mechanism of corrosion product film formation for the specimens in artificial seawater is illustrated in Figure 10. The formation of the corrosion product film was a result of electrochemical reactions occurring in the corrosive environment, and its structure and compactness directly influenced the material’s corrosion resistance [29]. Differences in the microstructure and mechanical properties of the specimens led to variations in the formation process and characteristics of the corrosion product films.

For the AC-1 and H-2 specimens, the lack of precipitates resulted in the formation of porous and non-uniform corrosion product films with numerous defects and pores. These imperfections served as pathways for the penetration of corrosive species (such as Cl^−^), thereby accelerating localized corrosion.

In contrast, the D-3 specimen contained dispersed Ni_3_Al within the matrix and a small amount of (Ni, Fe)_3_Al phases at the grain boundaries. The presence of these precipitates significantly improved the structure and compactness of the corrosion product film. The dispersed Ni_3_Al homogenized the micro-galvanic effect, reducing the likelihood of localized corrosion. Additionally, the high chemical stability and corrosion resistance of Ni_3_Al enabled it to generate Al_2_O_3_ during the corrosion process, filling defects in the Cu_2_O passive film and effectively blocking the penetration of Cl^−^ ions. Moreover, the excellent mechanical properties of the D-3 specimen allowed it to maintain a smooth surface under the impingement of artificial seawater, facilitating the formation of a flatter and more compact corrosion product film. The D-4 specimen had larger (Ni, Fe)_3_Al precipitates at the grain boundaries. Although these precipitates exhibited some corrosion resistance, their larger size led to selective corrosion and increased growth stress in the corrosion product film, resulting in crack formation. These cracks provided pathways for corrosive species to penetrate, reducing the protective effectiveness of the film. Therefore, while the corrosion product film of the D-4 specimen was more compact than that of the AC-1 and H-2 specimens, it still contained defects and did not match the protective performance of the D-3 specimen.

## 4. Conclusions

This study systematically investigated the microstructure, mechanical properties, and corrosion resistance of Cu-10Ni-3Al-1.8Fe-0.8Mn alloy under different heat treatments and drew the following conclusions:

1. The D-3 specimen, aged at 500 °C, exhibited the highest hardness (118 HV5), primarily due to the fine and uniformly dispersed Ni_3_Al precipitates in the matrix. These precipitates significantly enhanced the mechanical properties of the alloy through dispersion strengthening.
2. The D-3 specimen had the lowest erosion–corrosion rate of 0.0075 mm/a in artificial seawater. This was attributed to the fine and dispersed Ni_3_Al precipitates, which not only homogenized the micro-galvanic effect and reduced localized corrosion but also promoted the formation of a dense Cu_2_O oxide layer that effectively blocked the penetration of corrosive species.
3. The corrosion product film of the D-3 specimen was dense and featured a flat interface with the matrix, demonstrating the best protective performance. EIS analysis indicated that the D-3 specimen had the highest charge transfer resistance and corrosion product film impedance, further confirming its excellent corrosion resistance.
4. Deformation, followed by aging at 500 °C, is the optimal heat treatment process for Cu-10Ni-3Al-1.8Fe-0.8Mn alloy, significantly enhancing its mechanical properties and resistance to erosion–corrosion. This treatment is suitable for high-demand applications in marine environments.
5. This study can deepen the understanding of the relationship between the microstructure of copper–nickel alloys and the erosion–corrosion behavior. Long-term exposure experiments in real marine environments could be carried out in the future to assess the corrosion resistance and mechanical properties of copper–nickel alloys under real conditions.

## Figures and Tables

**Table 1:** Measured chemical composition of Cu-10Ni-3Al-1.8Fe-0.8Mn alloy (wt%).

	Ni	Al	Fe	Mn	Cu
Cu-10Ni-3Al-1.8Fe-0.8Mn	9.98	2.97	1.78	0.78	Bal.

**Table 2:** Alloy specimen numbers and heat treatments.

Specimen	Heat Treatment
AC-1	As-cast
H-2	Homogenization (950 °C/4 h)
D-3	Deformation–aging (500 °C/0.5 h)
D-4	Deformation–aging (750 °C/0.5 h)

**Table 3:** Fitting parameters of the equivalent circuit.

Specimen	AC-1	H-2	D-3	D-4
R_s_ (Ω·cm^2^)	23.49	1.77	10.56	42.24
R_ct_ (Ω·cm^2^)	441	9	1146	249
Q_ct_ × 10^−5^ (F·cm^−2^)	7.83	0.40	11.50	0.17
n_1_	0.78	0.69	0.99	0.90
R_f1_ (Ω·cm^2^)	12.35	33.25	1339	1117
Q_1_ × 10^−5^ (F·cm^−2^)	3.22	24.43	0.79	7.40
n_2_	0.77	0.61	0.95	0.82
R_f2_ × 10^3^ (Ω·cm^2^)	104.62	10.42	298.50	175.91
Q_2_ × 10^−5^ (F·cm^−2^)	8.22	38.53	16.18	0.52
n_3_	0.66	0.62	0.92	0.79
R_t_ × 10^3^ (Ω·cm^2^)	105.07	10.46	300.99	177.28
∑χ^2^ × 10^−3^	1.47	0.65	0.22	1.55

## Data Availability

The original contributions presented in this study are included in the article. Further inquiries can be directed to the corresponding author.
